# Correspondence

**Published:** 1881-09

**Authors:** 


### CORRESPONDENCE.

NASHVILLE, TENN., Aug. 8, 1881.

*Dr. J. Taft, Editor Dental Register, Cincinnati, Ohio*:

At a called meeting of the Nashville Dental Association held this afternoon at three o'clock, at the office of Drs. W. H. Morgan and Son, the following preamble and resolutions were adopted, relative to the death of Dr. J. H. Webber:

Death must come to *all* men, but when the summons comes to one in the prime and strength of manhood, engaged in the active duties of life, it is doubly solemn.

Our professional Brother, J. H. Webber, D. D. S., who departed this life at one o'clock this morning, was 40 years of age and in the midst of an useful life. He was kind, gentle and devoted to his profession, and we shall miss him in the busy walk of life, and especially at our monthly and annual meetings.

Resolved, That we humbly submit to this dispensation of the Divine Ruler of the world; that we will cherish and emulate his virtues.

Resolved, That we tender our sincere sympathies to the bereaved family, and pray the Divine benediction upon it.

Resolved, That we attend the funeral in a body, and that a copy of these resolutions be sent the bereaved family, also that a copy be furnished our city papers, and some of the different Dental Journals.

W. H. Morgan,
R. Russell,
A. T. Kline,
Committee.

